# Rapid sociometric mapping of community health workers to identify opinion leaders using an SMS platform: a short report

**DOI:** 10.1186/s13012-017-0611-y

**Published:** 2017-06-26

**Authors:** Thomas A. Odeny, Maya Petersen, Charles T. Muga, Jayne Lewis-Kulzer, Elizabeth A. Bukusi, Elvin H. Geng

**Affiliations:** 1 Center for Microbiology Research, Kenya Medical Research Institute, Nairobi, Kenya; 2 School of Public Health, University of California, Berkeley, Berkeley, CA USA; 3 Division of HIV/AIDS, Infectious Diseases and Global Medicine, Department of Medicine, San Francisco General Hospital, University of California, San Francisco, San Francisco, CA USA; 4 University of California, San Francisco, San Francisco, CA USA

## Abstract

**Background:**

Using opinion leaders to accelerate the dissemination of evidence-based public health practices is a promising strategy for closing the gap between evidence and practice. Network interventions (using social network data to accelerate behavior change or improve organizational performance) are a promising but under-explored strategy. We aimed to use mobile phone technology to rapidly and inexpensively map a social network and identify opinion leaders among community health workers in a large HIV program in western Kenya.

**Methods:**

We administered a five-item socio-metric survey to community health workers using a mobile phone short message service (SMS)-based questionnaire. We used the survey results to construct and characterize a social network of opinion leaders among respondents. We calculated the extent to which a particular respondent was a popular point of reference (“degree centrality”) and the influence of a respondent within the network (“eigenvector centrality”).

**Results:**

Surveys were returned by 38/39 (97%) of peer health workers contacted; 52% were female. The median survey response time was 13.75 min (inter-quartile range, 8.8–38.7). The total cost of relaying survey questions through a secure cloud-based SMS aggregator was $8.46. The most connected individuals (high degree centrality) were also the most influential (high eigenvector centrality). The distribution of influence (eigenvector centrality) was highly skewed in favor of a single influential individual at each site.

**Conclusions:**

Leveraging increasing access to SMS technology, we mapped the network of influence among community health workers associated with a HIV treatment program in Kenya. Survey uptake was high, response rates were rapid, and the survey identified clear opinion leaders. In sum, we offer proof of concept that a “mobile health” (mHealth) approach can be used in resource-limited settings to efficiently map opinion leadership among health care workers and thus open the door to reproducible, feasible, and efficient empirically based network interventions that seek to spread novel practices and behaviors among health care workers.

## Introduction

Using opinion leaders to accelerate the dissemination of novel, innovative, or evidence-based practices in health care is a promising strategy for closing the gap between evidence and practice. Sociologists have observed for a generation that new ideas and practices—from adoption of contraceptives to new farming practices—spread through social networks [1]. Social networks also play a role in the spread of behaviors among health care workers. A seminal study in diffusion science conducted in the 1950s and still cited today found that opinion leaders in a community of physicians drove uptake of a new antibiotic [2]. Since then, numerous studies have documented that social forces can promote uptake of novel diagnostic and treatment strategies [3–6]. In a Cochrane review of 18 randomized trials, the use of opinion leaders to improve the behavior of health workers led to an average of 12% increase in compliance with desired behavior as compared to no use of an opinion leader [7].

Network interventions, defined by Valente as “the process of using social network data to accelerate behavior change or improve organizational performance” (e.g., the use of opinion leaders to catalyze dissemination) are promising [8]. Research to test network-based interventions could potentially play an important role in the global treatment response to HIV [9, 10]. Yet, socio-metric surveys can be difficult to carry out repeatedly in all settings where an intervention is desired. Therefore, despite their promise, the feasibility of network-based interventions in resource poor settings is unclear.

Use of popular opinion leaders and sociometric peer leader selection strategies may improve service provider engagement in HIV care [11, 12]. In addition, the rapid increase in electronic communication and social media, especially mHealth applications, could enable more widespread use of network interventions [13]. In this short report, we describe a case study of application of mobile phone technology to map the network of social influence and identify opinion leaders with regard to professional behavior among a cadre of community health workers in Kenya where mobile phone networks are robust and cell phone ownership is common. This sociometric survey was carried out as part of a project that sought to promote more personalized counseling practices among peer counselors supporting people living with HIV. The larger project (not reported in this Short Report) will evaluate the effect of using this mobile phone-based sociometric survey on adoption of more personalized counseling practices among identified opinion leaders and on patient outcomes such as retention in care and HIV virologic suppression. This Short Report is focused on healthcare worker behavior that could potentially be addressed through opinion leaders.

## Methods

### Population

This study was conducted in May 2015 in three sub-counties in western Kenya: Kisumu (urban), Migori (semi-rural), and Rongo (semi-rural). Eligible participants were all lay peer health workers, also called clinical and community health assistants (CCHA), associated with the Family AIDS Care and Education Services (FACES) program in Kenya. FACES is a HIV prevention, care, and treatment program funded by the US President’s Emergency Plan for AIDS Relief (PEPFAR). It is a collaboration between the Kenya Medical Research Institute and the University of California, San Francisco [14]. At the time, FACES cared for over 80,000 patients and supported 132 government health facilities spread across western Kenya to offer HIV services. This region has the highest HIV prevalence in Kenya (15%) [15]. The participants were selected from four health facilities involved in a larger study on adaptive interventions including peer navigator approaches for improving engagement in HIV care (ClinicalTrials.gov #NCT02338739). We aimed to identify opinion leaders who would serve as referral contacts for CCHA peer navigator work. All CCHA at these facilities were invited to participate. The CCHA are lay healthcare workers unique to FACES trained as part of task shifting to provide peer counseling, HIV education, patient tracing, and other non-clinical or minor clinical tasks at health facilities that provide HIV care and treatment services [14]. While some of their tasks are similar to those of typical community health workers, they have no government-equivalent cadre due to the wide variety of tasks they are trained by FACES to carry out. They receive a monthly stipend from FACES of between $200 and $400 depending on experience and number on average five per health facility. A community liaison officer also employed by FACES oversees all the CCHA within a sub-county. In general, the CCHA collaborate across sub-counties by virtue of their patient tracing activities, which typically go beyond their assigned sub-county depending on patients’ location.

### Procedures

**Fig. 1:**
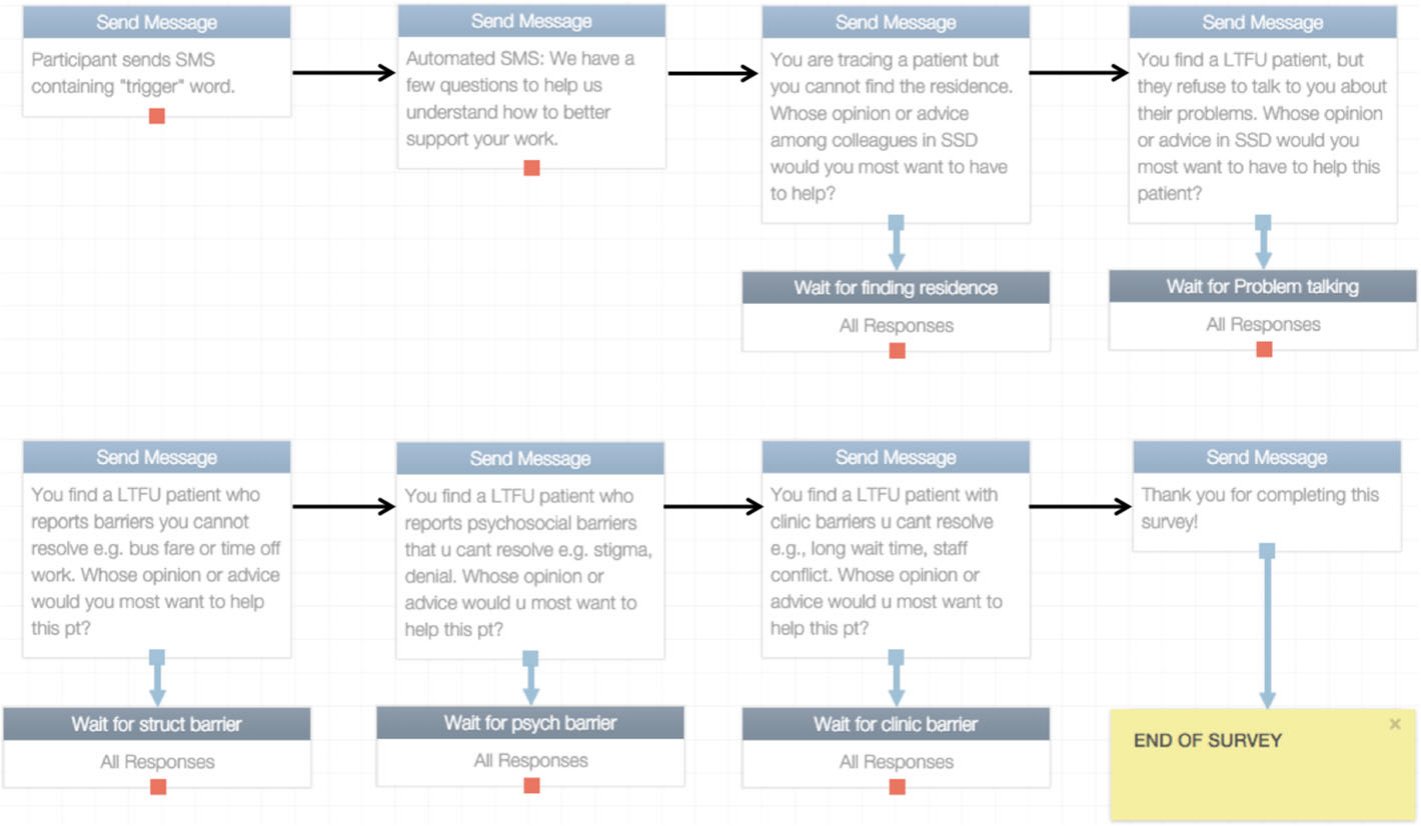
SMS survey flow illustration

We used an online open-source software platform to build an interactive survey delivered by short message service (SMS; www.RapidPro.io) [16]. This platform enables quick development and deployment of SMS surveys over a few hours by persons with no formal programming expertise. We administered this survey to the participants using an interactive mobile phone-based questionnaire (Table 1). In each sub-county, peer health workers were approached at the end of a didactic training session for a study on peer navigator approaches for improving engagement in HIV care. The participants sat in a group and were provided a list containing names of all CCHA in their sub-county and the FACES program-wide CCHA leadership, including the community liaison officers and overall CCHA department head. A code was assigned to each name on the list. The first item on the list was a code assigned to the name “no one” to cater for situations where respondents felt they would not need the opinion or advise of their colleagues. To begin the survey, the participants were asked to send a text message containing a unique “trigger” keyword. Upon receiving this keyword, an automated software system sent sequential questions (Table 1) via SMS to each participant with instructions to respond to each question by sending back an SMS containing a code representing a name on the list. When a response was successfully received by the automated system, the next question in the sequence was sent by SMS. A “thank you” SMS was sent to participants upon successful completion of the survey. The dialog-based SMS survey is illustrated in Fig. 1. The participants received mobile phone airtime credit worth 20 Kenyan shillings (~0.2 USD) to enable completion of the survey.

**Table 1:** Five-item network survey

1. You are tracing a patient but you cannot find the residence. Whose opinion or advice among colleagues in SSD would you most want to have to help?
2. You find a LTFU patient, but they refuse to talk to you about their problems. Whose opinion or advice in SSD would you most want to have to help this patient?
3. You find a LTFU patient who reports barriers you cannot resolve e.g. bus fare or time off work. Whose opinion or advice would you most want to help this pt?
4. You find a LTFU patient who reports psychosocial barriers that u cant resolve e.g. stigma, denial. Whose opinion or advice would u most want to help this pt?
5. You find a LTFU patient with clinic barriers u cant resolve e.g., long wait time, staff conflict. Whose opinion or advice would u most want to help this pt?

### Data analysis

To describe the process and efficiency of using the automated SMS system, we analyzed survey response rates, times, and costs using descriptive statistics. All descriptive analyses were done using STATA v13.0. We used the survey responses to construct a social network diagram. We measured the “centrality” of networks of relations between peer health workers using the network analysis package igraph in R v3.0.1. This analysis aimed at identifying connections among peer health workers as well as revealing gaps in their professional relations. Specifically, we calculated (1) the extent to which a particular peer health worker was a popular point of reference (“degree centrality”) as indicated by the number of connections with other members of the network and (2) the influence or importance of a peer health worker within the network as indicated by the extent to which a peer health worker was connected to other connected peer health workers (“eigenvector centrality”). Both measures of centrality were calculated based on a combination of all the survey questions. The opinion leader was defined as one with the highest eigenvector centrality.

### Ethical considerations

This survey was conducted as part of a large study (ClinicalTrials.gov #NCT02338739) focused on adaptive retention interventions including a peer navigator intervention. The study protocol was approved by the Scientific and Ethical Review Unit of the Kenya Medical Research Institute and University of California San Francisco.

## Results

We invited a total of 39 peer health workers to participate, all of whom accepted to complete the surveys: 14 (36%) from Kisumu sub-county, 9 (23%) from Rongo sub-county, and 16 (41%) from Migori sub-county. Participant demographics are summarized in Table 2. Most participants were female (52%), and all had higher than secondary level of education. The median age of peer health workers was 31 years (inter-quartile range (IQR) 27–37), and the median duration on the job was 3 years (IQR 0.5–7).

**Table 2:** Participant demographic characteristics

	Kisumu	Migori	Rongo
	(*n* = 21)	(*n* = 28)	(*n* = 14)
Characteristic			
Age, median years (IQR)	32 (29–38)	28 (26–35)	32.5 (28–40)
Years worked as CCHA, median (IQR)	7 (0.6–8)	2 (0.3–5)	3.5 (0.5–7)
Female, *n* (%)	12 (57)	17 (61)	4 (29)
Post-secondary education, *n* (%)	21 (100)	28 (100)	14 (100)

*IQR* inter-quartile range, *CCHA* clinic and community health assistant

**Fig. 2:**
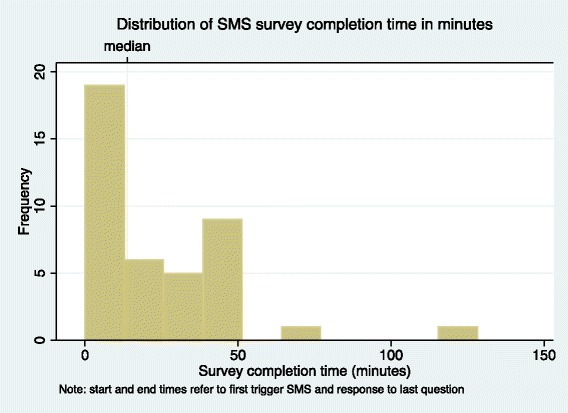
Distribution of SMS survey completion times

**Fig. 3:**
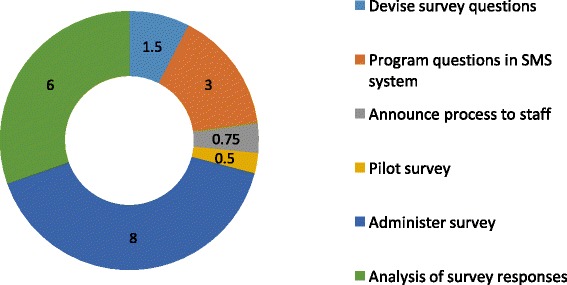
Distribution of time spent (in hours) on survey development tasks per sub-county. The total time spent was 19.75 h distributed as follows: (1) devise survey questions 1.5 h (8%); (2) program questions in SMS system 3 h (15%); (3) announce process to staff 0.75 h (4%); (4) pilot survey 0.5 h (3%); (5) administer survey 8 h (40%); and (6) analysis of survey questions 6 h (30%)

A total of 39 surveys (285 survey questions) were sent by SMS. Of these, 38/39 surveys were completed (97%). The participants sent a total of 282 SMS responses. The median survey response time (defined as time from sending initial “trigger” SMS to receiving “thank you” SMS upon survey completion) was 13.75 min (IQR, 8.8–38.7 min) (Fig. 2). The distribution of time spent on each component of the survey within one sub-county is shown in Fig. 3. Each SMS response from a participant cost 1 Kenyan shilling (~0.01 USD) for a total cost of 282 Kenyan shillings (~2.82 USD). Each incoming and outgoing SMS routed through our automated SMS system cost 1 US cent to relay through a secure cloud-based SMS aggregator, for a total cost of 2.85 USD for outgoing survey SMS and 2.82 USD for incoming response SMS. The sum total of sending survey questions and receiving SMS responses was US$ 8.46.

**Fig. 4:**
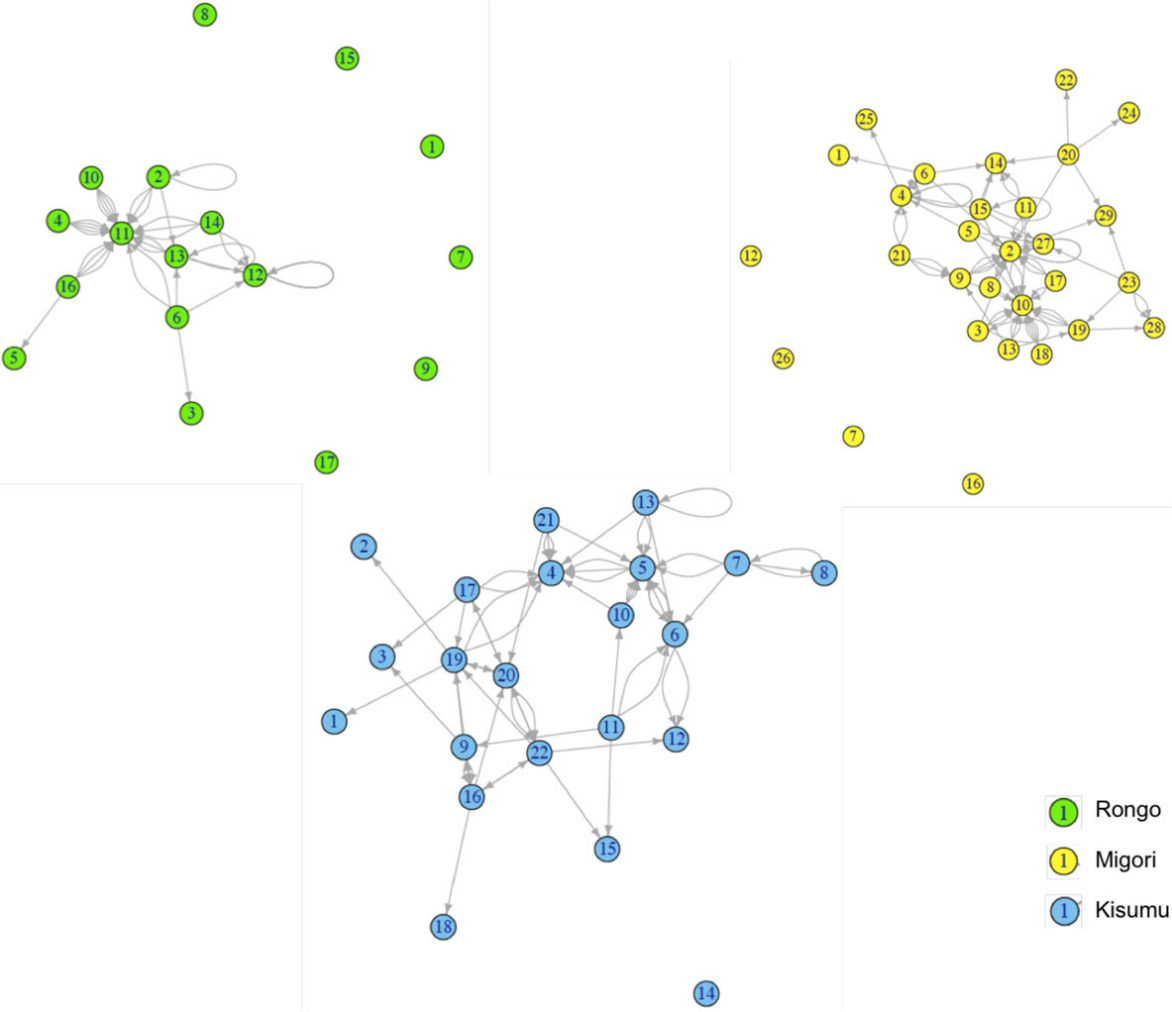
Network structure visualization. Each *node* represents a survey respondent, and each *arrow* points to the person whose opinion would be sought in response to a survey question. Each *arrow* represents one survey question. An *arrow* pointing back to the *node* of origin represents a survey respondent who, in response to a question asking whose opinion they would seek, indicated that they would rely on their own opinion. *Isolated nodes* (no incoming or outgoing arrows) represent respondents who neither selected others as a reference for opinions nor selected themselves

**Fig. 5:**
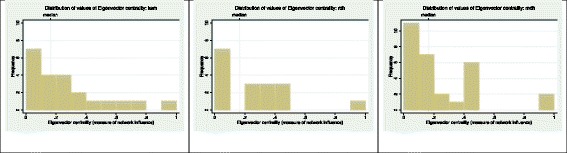
Distribution of eigenvector centrality values. *KSM* Kisumu sub-county, *RDH* Rongo sub-county, *MDH* Migori sub-county

The network connections between peer health workers are visually represented in Fig. 4. Networks were dense at each sub-county, i.e., there was a high number of connections as a proportion of the maximum possible number of connections. Individuals identified as the most connected (high degree centrality) were also the most influential (high eigenvector centrality). The distribution of influence (eigenvector centrality) was highly skewed in favor of a single influential individual at each site as depicted by the large gap between the highest eigenvector value and the remaining values in Fig. 5. Of the top five influential individuals overall, only two held supervisory positions over other peer health workers.

## Discussion

Leveraging the increasing access to SMS technology, we mapped the network of influence in a cadre of peer health workers associated with a HIV care and treatment program in Kenya. Using mobile phones, we identified opinion leaders by carrying out a sociometric survey in Kenya over the course of 3 days and at a cost of 8.46 USD.

This work represents a novel application of mobile phone technology to rapidly reveal network characteristics and identify influential individuals in a cadre of peer health workers, with direct implications for network interventions in global health. To our knowledge, this is the first ever application of mobile phone technology to do a sociometric survey to identify health care worker opinion leaders in Africa. Mobile phone penetration into the general public has exceeded 80% in Kenya, and in the health care workforce, it is estimated to be 100%, with 99% of health workers using SMS [17]. Indeed, despite the fact that peer health workers are a relatively low-paid cadre, all peer health workers included in this study had a personal mobile phone. Overall, 97% of participants completed the survey—indicating high acceptance and feasibility. The rapidity of the exercise demonstrates the relative ease of a survey delivered over the mobile phone platform, which allows a large number of respondents to be reached and respond despite being geographically dispersed. This is especially relevant for health workers in resource-limited settings where health facilities are widely dispersed and the terrain often difficult to navigate to bring groups of people together. The short time taken also meant that the survey could be conducted without placing an extra time burden on the already strained system.

Efficient mapping of social networks opens the door to reproducible, feasible, and efficient empirically based network interventions that seek to spread novel practices and behaviors among health care workers [9]. We found clearly influential individuals within a network of peer health workers in Kenya. The existence of influential persons, if widespread, can potentially be leveraged to catalyze dissemination of novel practices. Opinion leaders have been used in many settings as agents of behavior change in their communities. In this survey, we found a clear hierarchy of influence as measured by eigenvector centrality. This asymmetry indicates that it may be possible to target specific individuals to achieve change on a larger scale. Interventions can be more efficient if training, teaching, or capacitation is targeted toward a few individuals. Interventions can be more effective if the leaders of change will bring the community along with them through informal leadership. Of note, the persons of influence did not always coincide with positions of official authority—of the top five persons identified, only two held positions of overall authority, suggesting that informal network relations are crucial and must be measured empirically to be identified. Administrative or formal authority is an imperfect proxy of real influence in the network of health workers in Kenya.

This study had a number of limitations. First, the network mapping was not comprehensive in that we limited responses to only one “influencer” per topic. It would have been feasible to include more than one influencer and thereby have a denser network, but we sought to restrict our analysis to identify the top most influential individual. Second, a number of individuals reported no one as the answer to the questions posed. This could be interpreted in a number of ways. For example, it may indicate that they are otherwise confident in their own abilities to tackle the problem or it may also mean that the person they would turn to was not among the group of peer health workers included in the study. Third, the results on feasibility of the approach may not apply to other cadres and other settings. In our context, the peer health workers were paid by a PEPFAR-funded program and were approached following a training supported by a research study. Fourth, while the survey questionnaire was delivered using SMS, the survey respondents still met physically at one place and a paper list was used with names and codes to aid survey completion. We could potentially have delivered the entire survey using SMS without having the respondents meet physically. Of note, peer health workers in this region have periodic in-person meetings (sometimes as frequent as weekly) to review work plans and share experiences. We leveraged these already existing meetings to achieve high survey response and completion rates. However, we believe that the questionnaire can be delivered entirely using mobile phone technology without a physical meeting. Finally, we used a convenience sample, which may lead to biased responses.

## Conclusions

In conclusion, we demonstrate the use of SMS technology using open-source software to carry out a rapid sociometric survey in Kenya among a cadre of community health workers. The rapid rise in access to mobile technology in resource-limited settings can be leveraged to efficiently conduct sociometric mapping among health workers at low cost.

## Abbreviations

CCHA: Clinical and community health assistants
FACES: Family AIDS Care and Education Services
mHealth: Mobile health
PEPFAR: President’s Emergency Plan for AIDS Relief
